# Commentary: Rapid Phosphoproteomic Effects of Abscisic Acid (ABA) on Wild-Type and ABA Receptor-Deficient *A. thaliana* Mutants

**DOI:** 10.3389/fpls.2016.01062

**Published:** 2016-07-19

**Authors:** Xiaolin Wu, Wei Wang

**Affiliations:** State Key Laboratory of Wheat and Maize Crop Science, Collaborative Innovation Center of Henan Grain Crops, College of Science, Henan Agricultural UniversityZhengzhou, China

**Keywords:** abiotic stresses, abscisic acid, phosphoproteome, ABA signaling, *Arabidopsis thaliana*, proteomics

The plant hormone abscisic acid (ABA) plays an important role in regulating different stages of plant growth and development. It performs a broad range of essential functions in plant adaptation to abiotic stresses, particularly drought, elevated temperature, and salinity (Ahuja et al., 2010). Under abiotic stress, ABA regulates various sets of stress-inducible genes to initiate the synthesis of diverse proteins. Therefore, the engineering of the ABA signaling pathway can potentially offer new avenues for improving abiotic tolerance in plants. Over the prior two decades, the individual steps of the ABA signaling pathway, which range from signal perception to the nuclear action of downstream gene regulation, have been dissected (Yoshida et al., 2014). In particular, increasing evidence has revealed the central role of protein phosphorylation and dephosphorylation in ABA signaling in *Arabidopsis thaliana* (Kline et al., 2010; Umezawa et al., 2013; Wang et al., 2013) and crop plants (e.g., rice, wheat, and maize) (He and Li, 2008; Zhang et al., 2014; Hu et al., 2015) after lengthy ABA treatments (15 min or longer). *Arabidopsis* represents the most successful system for understanding protein phosphorylation. However, relatively little is known regarding the initial protein phosphorylation changes induced by ABA in plants. The identification of early ABA-inducible phosphoproteins using phosphoproteomic technologies should reveal novel and potentially key components in ABA signal transduction.

A recent study on the rapid phosphoproteomic effects of ABA on *A. thaliana* (Minkoff et al., 2015) provided critical insight into the regulatory mechanisms for several ABA-inducible phosphoproteins. A loss-of-function quadruple (pyr1; pyl1; pyl2; pyl4) receptor mutant of *A. thaliana* (QR) exhibits strong ABA insensitivity (Park et al., 2009). Using QR and wild-type (WT) plants as a model, Minkoff et al. (2015) performed a quantitative phosphoproteomic analysis to identify early (within 5 min) ABA-induced protein phosphorylation events mediated by ABA receptors (i.e., PYR1, PYL1, PYL2, and PYL4). After conducting the TiO_2_ enrichment of phosphopeptides, these authors used a combination of untargeted high-resolution MS and targeted MS to quantify a panel of phosphopeptides related to ABA signaling, and discuss their biological significance. The two most enriched phosphorylation motifs in the set of ABA-responsive phosphorylation were those of the SnRK2 and MAPKs, implicating these two pathways as the earliest activated in response to ABA stimulation. Furthermore, relative to the ABA-dependent phosphorylation changes observed in WT plants, different responses to ABA were observed in QR mutants: the majority of ABA-dependent phosphorylation changes were diminished, whereas many smaller ABA-dependent phosphorylation changes were not detected in the quadruple mutant plant. The reduced phosphorylation is consistent with the decreased ABA reception and signal in the mutant. Future studies need to elucidate the details regarding each of the PYR/PYL/RCAR receptors and their separate roles within the ABA-regulated phosphorylation network. Moreover, the functional importance of the dephosphorylation of ABA-responsive proteins remains to be determined.

It is becoming increasingly clear that the analysis of signaling networks is required to understand the dynamic and complex mechanisms that underlie cellular functions and outputs. In particular, research has demonstrated that kinase pathways become active within minutes—sometimes seconds—of a stimulus, even in crop plants (Schulze et al., 2010). At 15 and 30 min after the onset of a stress (e.g., drought, cold, or ABA), there are significant initial transcriptional changes in *Arabidopsis* (Kilian et al., 2007). Therefore, it is necessary to analyze the initial ABA signal perception and adaptation that occur within the first few minutes of ABA exposure in plants. Minkoff et al. (2015) identified multiple ABA-induced phosphorylation changes that occur within 5 min of ABA treatment, including three SnRK2 autophosphorylation events and the phosphorylation of SnRK2 substrates. However, the detection and quantification of transient and weak ABA signals at shorter ABA treatment time points (e.g., 1 min) may represent a challenge for future research.

Importantly, Minkoff et al. (2015) analyzed the rapid phosphoproteomic effects of ABA, primarily in *A. thaliana* leaves. Root tips have long been regarded as the main sites of ABA biosynthesis, and synthesized ABA is transported to target tissues to induce responses (Koiwai et al., 2004). Given the importance of root tips (e.g., the root cap) in simultaneously sensing and responding to moisture (Eapen et al., 2005), the further dissection of specific rapid phosphoproteomic effects of ABA in *A. thaliana* roots will provide a more comprehensive understanding of ABA signaling.

The comprehensive and intensive work of Minkoff et al. (2015) supports hypotheses regarding ABA signaling and provides critical insights with respect to the ABA mode of action by which the family of ABA receptors directs rapid phosphoproteomic changes. The more interesting aspect is that the conserved ABA signaling mechanisms in plant could lead to improved understanding of ABA signaling in crop plants, and help design plant traits to improve economic productivity. Furthermore, the methodology of phosphoproteomics study in ABA for Arabidopsis could be applied to studies of phospho-signalings for other phytohormones in Arabidopsis and other crop plants.

## Author contributions

XW drafted the paper. WW edited the paper.

## Funding

Funding for open access charge: International Science and Technology Cooperation and Exchange Project of Zhengzhou Municipal Science and Technology Bureau (153PGJHZ207), Henan, China.

### Conflict of interest statement

The authors declare that the research was conducted in the absence of any commercial or financial relationships that could be construed as a potential conflict of interest.

## References

[B1] Ahuja I. de Vos R. C. H. Bones A. M. Hall R. D. (2010). Plant molecular stress responses face climate change. Trends Plant Sci. 15, 664–674. 10.1016/j.tplants.2010.08.002 20846898

[B2] Eapen D. Barroso M. L. Ponce G. Campos M. E. Cassab G. I. (2005). Hydrotropism: root growth responses to water. Trends Plant Sci. 10, 44–50. 10.1016/j.tplants.2004.11.004 15642523

[B3] He H. Li J. (2008). Proteomic analysis of phosphoproteins regulated by abscisic acid in rice leaves. Biochem. Bioph. Res. Commun. 371, 883–888. 10.1016/j.bbrc.2008.05.001 18468508

[B4] Hu X. L. Li N. N. Wu L. J. Li C. Q. Li C. H. Zhang L.. (2015). Quantitative iTRAQ-based proteomic analysis of phosphoproteins and ABA-regulated phosphoproteins in maize leaves under osmotic stress. Sci. Rep. 5:15626. 10.1038/srep15626 26503333 PMC4650667

[B5] Kilian J. Whitehead D. Horak J. Wanke D. Weinl S. Batistic O.. (2007). The AtGenExpress global stress expression dataset: protocols, evaluation and model data analysis of UV-B light, drought and cold stress responses. Plant J. 50, 347–363. 10.1111/j.1365-313X.2007.03052.x 17376166

[B6] Kline K. G. Barrett-Wilt G. A. Sussman M. R. (2010). In planta changes in protein phosphorylation induced by the plant hormone abscisic acid. Proc. Natl. Acad. Sci. U.S.A. 107, 15986–15991. 10.1073/pnas.1007879107 20733066 PMC2936636

[B7] Koiwai H. Nakaminami K. Seo M. Mitsuhashi W. Toyomasu T. Koshiba T. (2004). Tissue-specific localization of an abscisic acid biosynthetic enzyme, AAO3, in Arabidopsis. Plant Physiol. 134, 1697–1707. 10.1104/pp.103.036970 15064376 PMC419843

[B8] Minkoff B. B. Stecker K. E. Sussman M. R. (2015). Rapid phosphoproteomic effects of abscisic acid (ABA) on wild-type and ABA receptor-deficient A. thaliana mutants. Mol. Cell. Proteomics 14, 1169–1182. 10.1074/mcp.M114.043307 25693798 PMC4424391

[B9] Park S. Y. Fung P. Nishimura N. Jensen D. R. Fujii H. Zhao Y.. (2009). Abscisic acid inhibits type 2C protein phosphatases via the PYR/PYL family of START proteins. Science 324, 1068–1071. 10.1126/science.1173041 19407142 PMC2827199

[B10] Schulze B. Menzel T. Jehle A. K. Müller K. Beeler S. Boller T.. (2010). Rapid heteromerization and phosphorylation of ligand-activated plant transmembrane receptors and their associated kinase BAK1. J. Biol. Chem. 285, 9444–9451. 10.1074/jbc.M109.096842 20103591 PMC2843194

[B11] Umezawa T. Sugiyama N. Takahashi F. Anderson J. C. Ishihama Y. Peck S. C.. (2013). Genetics and phosphoproteomics reveal a protein phosphorylation network in the abscisic acid signaling pathway in *Arabidopsis thaliana*. Sci. Signal. 6:rs8. 10.1126/scisignal.2003509 23572148

[B12] Wang P. Xue L. Batelli G. Lee S. Hou Y. J. van Oosten M. J.. (2013). Quantitative phosphoproteomics identifies SnRK2 protein kinase substrates and reveals the effectors of abscisic acid action. Proc. Natl. Acad. Sci. U.S.A. 110, 11205–11210. 10.1073/pnas.1308974110 23776212 PMC3703982

[B13] Yoshida T. Mogami J. Yamaguchi-Shinozaki K. (2014). ABA-dependent and ABA-independent signaling in response to osmotic stress in plants. Curr. Opin. Plant Biol. 21, 133–139. 10.1016/j.pbi.2014.07.009 25104049

[B14] Zhang M. Lv D. W. Ge P. Bian Y. W. Chen G. X. Zhu G. R.. (2014). Phosphoproteome analysis reveals new drought response and defense mechanisms of seedling leaves in bread wheat (*Triticum aestivum* L.). J. Proteomics 109, 290–308. 10.1016/j.jprot.2014.07.010 25065648

